# Potential of Chickpea Flours with Different Microstructures as Multifunctional Ingredient in an Instant Soup Application

**DOI:** 10.3390/foods10112622

**Published:** 2021-10-29

**Authors:** Laura E. C. Noordraven, Hyun-Jung Kim, Hans Hoogland, Tara Grauwet, Ann M. Van Loey

**Affiliations:** 1 KU Leuven, Laboratory of Food Technology, Department of Microbial and Molecular Systems (M2S), Kasteelpark Arenberg 23, P.O. Box 2457, 3001 Leuven, Belgium; laura.noordraven@kuleuven.be (L.E.C.N.); tara.grauwet@kuleuven.be (T.G.); 2 Unilever Foods Innovation Centre, Department of Product & Process Science Foods, Bronland 14, 6708 WH Wageningen, The Netherlands; hyun-jung.kim@unilever.com (H.-J.K.); hans.hoogland@unilever.com (H.H.)

**Keywords:** chickpea flour, rheology, gas chromatography-mass spectrometry fingerprinting, powder flowability, instant soup

## Abstract

Chickpea flours are an interesting multifunctional ingredient for different food products. This study investigated the potential of differently processed chickpea flours as alternative thickening agents in an instant soup recipe, replacing potato starch. Dry instant soup powders were compared on bulk density and powder flowability, whereas prepared liquid instant soups were studied in terms of rheological behaviour (as influenced by microstructure) and volatile composition. The chickpea-flour-containing soup powders possessed similar powder flowability to a reference powder but were easier to mix and will potentially result in reduced blockages during filling. For prepared liquid instant soups, similar viscosities were reached compared to the potato starch reference soup. Nevertheless, the chickpea-flour-containing soups showed higher shear thinning behaviour due to the presence of larger particles and the shear induced breakdown of particle clusters. Flavour compounds from the soup mix interacted with chickpea flour constituents, changing their headspace concentrations. Additionally, chickpea flours introduced new volatile compounds to the soups, such as ketones, aldehydes, alcohols, and sulphur compounds, which can possibly alter the aroma and flavour. It was concluded that chickpea flours showed excellent potential as alternative thickening ingredient in instant soups, improving the protein, mineral and vitamin content, and the powder flowability of the soups, although the flavour of the soups might be affected by the changes in volatile profiles between the soups.

## 1. Introduction

Chickpeas are a healthy and sustainable food source that could be processed into an interesting multifunctional ingredient to be used in different types of food applications. Several studies have shown that suspended desi and kabuli chickpea flours showed viscosifying potential upon heating. It was reported that cultivar, thermal conditions, cell intactness, and starch state all significantly influenced the rheological properties of these chickpea flours in aqueous systems, using both hot and cold swelling treatments [1,2,3,4,5,6]. However, these studies were carried out using simple water-flour suspensions. When chickpea flour would be added in a food application, other food ingredients would be present that may influence the thickening capacity of the chickpea flours. Lipids, for example, can coat starch granules and therefore reduce the viscosifying properties of starch due to a limitation in water uptake [7]. Additionally, cell wall material is reported to reduce the viscosifying properties of rice flour, whereas in cow pea flour it resulted in an increased viscosifying potential [8,9,10].

Instant soup is a typical semi-liquid food application which contains relatively high amounts of starch. It is a popular food in modern society, due to its minimal preparation time, relatively long shelf-life, and its light weight, which makes it easy to transport [11,12]. To improve the nutritional profile of instant soups, legume and vegetable powders could be added [12]. As commercially available instant soups often contain substantial amounts of isolated potato or corn starch, a replacement of the starch with chickpea flour could improve the nutritional value of the product. Chickpea flours are, for example, rich in the essential amino acids leucine, lysine, and phenyl alanine, and the non-essential amino acids glutamic acid, aspartic acid, and arginine, but lack high amounts of sulphur containing amino acids methionine and cysteine [13]. Therefore, they could be an excellent addition to a grain based diet, which is higher in sulphur containing amino acids but low in lysine, to meet the daily amino acid requirements [13,14]. Moreover, chickpeas are an important source of vitamins and minerals. They generally contain calcium, magnesium, iron, and zinc [14]. Chickpeas mainly contain water-soluble vitamins (e.g., folic acid (B9), riboflavin (B2), pantothenic acid (B5), pyridoxine (B6), and vitamin C). Furthermore, γ tocopherol and α-tocopherol (vitamin E), vitamin A, and vitamin K are present in chickpeas [13,14]. However, thus far, research on the behaviour of chickpea flours as a thickening agent in (semi-)liquid food products is scarce.

A benefit of industrially used starches is that they often do not largely affect the flavour of food products when added in low concentrations [15]. The addition of chickpea flour on the other hand, could possibly influence sensory properties such as the colour, aroma, and flavour, apart from the desired viscosity changes. Limited research on the volatile profile of raw and roasted chickpea flours, as well as soaked, germinated, cooked, and sterilised whole chickpeas has been reported. These studies indicate that processing methods influences the volatile profile of chickpeas [16,17,18,19,20,21]. However, the odour threshold and concentration of the volatiles in chickpea flour, as well as interactions of the volatiles with each other and with other food ingredients such as proteins, starch, and lipids, determine the final perception of the aroma and flavour of chickpea-flour-containing products [22,23,24,25,26,27].

In this context, the present study aimed to investigate the potential of chickpea flours with different microstructures as a replacement of potato starch in an industrially relevant instant soup recipe. Dry instant soup powders and prepared liquid instant soups were analysed, including the characterisation of bulk density, particle size distribution, microstructure, and the rheological behaviour of powders and soups. Additionally, the impact of the replacement of potato starch with chickpea flour on the volatile profile of the soups was investigated.

## 2. Materials and Methods

### 2.1. Materials

Frozen dried kabuli chickpeas (*Cicer arietinum* L.), harvested in Argentina in December 2018, were supplied by Greenyard Prepared (Bree, Belgium). The chickpeas were stored at −40 °C until usage. A commercially available pre-gelatinised chickpea flour (Instant Hummus Powder (INS)) was obtained from Codrico (Rotterdam, The Netherlands). All other soup ingredients were provided by Unilever (Wageningen, The Netherlands).

### 2.2. Preparation of the Flours

Non-gelatinised open cell chickpea flour (NG-O) and pre-gelatinised open cell chickpea flour (PG-O) were prepared as described by Noordraven et al. (2021) [6] with minor adjustments. NG-O was made by consecutively soaking the frozen raw chickpeas overnight in demineralised water at 25 °C, removing the seed coat, and mashing the chickpeas in their own soaking water for 5 min using an Ultra-Turrax T25 (Janke & Kunkel, IKA Labortechnik, Staufen, Germany) at 4000 rpm, resulting in a puree with open cells. This puree was lyophilised using a FreeZone 12 L Freeze Dry System connected to a FreeZone Stoppering Tray Dryer Freeze System (Labconco Corporation, Kansas City, MO, USA) for 48–72 h and afterwards sieved (35 mesh (500 μm)) to obtain the NG-O flour. PG-O was obtained by soaking the raw chickpeas overnight in demineralised water at 25 °C, afterwards cooking the chickpeas in their soaking water for 50 min at 95 °C followed by a dehulling step. The chickpeas were made into a puree in their cooking water, which was lyophilised and sieved (35 mesh (500 μm)). The sieved pre-gelatinised flour was afterwards ball-milled (FRITSCH GmbH, pulverisette 6, Idar-Oberstein, Germany) for 5 min at 500 rpm to obtain open cells.

### 2.3. Instant Soup Preparation

Dry soup recipes with three different flour concentrations (low, medium, and high) were prepared as described in Table 1. The reference soup with potato starch (PS) was only prepared at the medium (reference) concentration. The soup base consisted of sugar, salt, yeast extract, palm oil, chicken base, onion powder, coconut flavouring, and several spices including lemon, soy sauce, ginger, cayenne pepper, turmeric, and coriander seeds and leaves. Boiling water was added to dry soup powder (ratio powder: water 1:10) and stirred for 2 min using a magnetic stirrer to obtain the liquid soup. For all analytical tests, soups containing NG-O, PG-O, and INS, as well as the reference soup containing PS (REF) were used.

### 2.4. Flow Behaviour of Instant Soups

Rheological measurements were performed using a stress-controlled rheometer (MCR 302, Anton Paar, Graz, Austria) with a starch stirrer cell (ST24–2D/2V/2V-30). Soups were prepared as described in Section 2.3, and 40 mL of soup was added to a concentric cylinder cup (3971 CC27). The temperature was set at 60 °C for the whole measurement, as this was the temperature of the soup directly after preparation. The cup was covered to avoid evaporation of the sample during the measurement. Samples were stabilised for 500 s at a shear rate of 1 s^−1^ and afterwards a shear rate sweep was performed by increasing the shear rate logarithmically from 1 to 250 s^−1^. Each shear rate was applied until steady-state viscosity was reached, with a maximum measuring time of 85 s per shear rate and 3 measuring points were taken per decade. All samples were measured in duplicate.

### 2.5. Powder Flowability

The powder flowability properties of the dry soup powders were analysed using a FT4 powder rheometer (Freeman Technology, Tewkesbury, United Kingdom) as described by Leturia et. al. (2014) [28], using the standard methods of the FT4 powder rheometer: the shear cell test and the stability and variable flow rate test. All samples were measured in duplicate.

The shear cell test was used to determine powder flowability when powder flow is initiated in high shear applications [28]. In this test, the powder was brought to a critically consolidated state by consecutively conditioning the powder with a dynamic blade to obtain a homogenous powder, pre-compacting the powder using a vented piston under a normal load of 3 kPa and finally conducting a pre-shear step. The shear stress necessary to create powder flow was afterwards measured at different normal loads. With this test the major principal stress (σ_1_) in Pa, which is the consolidation stress [29], and the unconfined yield strength (σ_c_) in Pa, defined as the maximum stress beyond which deformation of the consolidated material occurs [29,30], were determined. Flowability index (ff_c_) was calculated using Equation (1) [31].

ff_c_ = σ_1_/σ_c_ (1)

The stability and variable flow rate test consisted of flow energy measurements of seven identical tests, with a conditioning cycle in between every test, followed by four tests at reducing blade tip speeds, to measure the sensitivity of a powder for different flow rates [32]. Using this test, the basic flow energy (BFE) in J/g, specific energy (SE) in J/kg, stability index (SI), and flow rate index (FRI) of the powders were determined.

### 2.6. Apparent Bulk Density

Apparent bulk densities were determined using a manual bulk density tester (SMG 697, ERWEKA GmbH, Langen, Germany). A funnel was filled with powder and afterwards the powder was tapped into a tared receiver of 500 mL and weighed. The measurements were done in triplicate and the apparent bulk density was calculated as the powder weight per unit of volume (g/mL).

### 2.7. Particle Size Distribution

The particle size distribution was analysed using a Mastersizer 2000 particle size analyser with Hydro 2000s liquid module (Malvern Instruments Ltd., Malvern, UK). Potato starch, chickpea flours, and soup powders were suspended in cold or boiling demineralised water, stirred for 2 min using a magnetic stirrer, and injected to the system (obscuration range between 10 and 20%). The sample was pumped through the measuring cell (pump speed 1500 rpm), where particle light scattering took place. All samples were analysed without sonication, but the soup powders suspended in boiling water were additionally analysed with sonication (100%). Every sample was measured in duplicate and three measurements per sample were taken.

### 2.8. Light Microscopy

Potato starch and chickpea flours were suspended in cold and boiling demineralised water for microscopic observation. A Morphologi light microscope (Malvern Instruments Ltd., Malvern, United Kingdom) was used to observe the microstructures at 20× magnification and the Morphologi software (Malvern Instruments Ltd., Malvern, UK) was used to capture micrographs.

### 2.9. HS-SPME-GC-MS Volatile Fingerprinting

Untargeted headspace solid-phase microextraction-gas chromatography-mass spectrometry (HS-SPME-GC-MS) fingerprinting was used to analyse the headspace volatiles of the different soups and suspensions of the different chickpea flours and potato starch in boiling demineralised water (ratio powder to water 1:10), as described by Kebede et al. (2014) and Vervoort et al. (2012) [33,34], with adjustments to the GC-MS analysis.

For all flour suspensions and soup samples, 3 ± 0.05 g was added to glass vials (20 mL, Macherey-Nagel, Düren, Germany) and 3 mL saturated NaCl solution was added. During the whole sample preparation, samples remained in an ice bath to reduce the loss of volatiles. Afterwards, samples were vortexed and stored in the cooler tray (10 °C) of the autosampler (MPS, Gerstel GmbH, Mühlheim a/d Ruhr, Germany) of the GC-system until analysis.

A 7200 Accurate-Mass gas chromatography-quadrupole-time-of-flight-mass spectrometer (GC-Q-TOF-MS) (Agilent Technologies, Santa Clara, CA, USA) was used to analyse the volatile fraction of the samples. Per sample, six replications were analysed. The incubation and extraction temperatures were set at 50 °C (approximate consumption temperature of the soup) under agitation (250 rpm) for 5 and 20 min, respectively. Headspace volatiles were extracted using a 50/30 µm divinylbenzene/carboxen/polydimethylsiloxane (DVB/CAR/PDMS) Stable Flex fibre (Supelco, Bellefonte, PA, USA). Desorption of volatiles took place at the injection port of the GC for 5 min at 230 °C. Injection was performed in a splitless mode. Volatile separation was executed on a capillary TR-FFAP column (30 m × 250 μm × 0.25 μm, ThermoFisher, Waltham, MA, USA) with helium as the carrier gas at a constant flow of 1 mL/min and a pressure of 46.73 kPa. The temperature profile of the GC oven involved a holding step at 35 °C for 5 min, followed by a heating step to 230 °C at 5 °C/min, and finally a holding step at 230 °C for 1 min.

MS detection was obtained by electron ionization mode at 70 eV with a scanning range of 30−300 *m*/*z* and a scanning speed of 3 scans per second. The MS ion source and quadrupole temperatures were 230 and 150 °C, respectively.

### 2.10. Data Analysis

Statistical analysis for the apparent bulk density, powder flowability, and soup flowability was performed using the software JMP Pro 14.2 (SAS Institute, Cary, NC, USA). To test for significant differences, a one-way analysis of variance (ANOVA) followed by a multiple comparison Tukey HSD test (*p* < 0.05) was conducted. In case of unequal variances, a Welch’s test was used to determine if significant differences were present. Multivariate data analysis (MVDA) was performed on the GC-MS data. Pre-processing of GC-MS data, partial least squares discriminant analysis (PLS-DA) and the calculation of discriminant compounds (variable identification coefficients (VID)) were achieved as described by Kebede et al. (2014) and Vervoort et al. (2012) [33,34], using Automated Mass Spectral Deconvolution and Identification System (AMDIS) (version 2.72, 2014, National Institute of Standards and Technology, Gaithersburg, MD, USA) for peak deconvolution, Mass Profile Professional (MPP) (version B12.00, 2012, Agilent Technologies, Diegem, Belgium) for peak filtering and alignment, SOLO (version 8.7.1, 2020, eigenvector Research, Inc., Manson, WA, USA) for PLS-DA analysis, and OriginPro (OriginPro 2020, OriginLab Corporation, Northampton, MA, USA) for data visualisation.

## 3. Results and Discussion

### 3.1. Swelling Behaviour of the Soups

**Figure 1:**
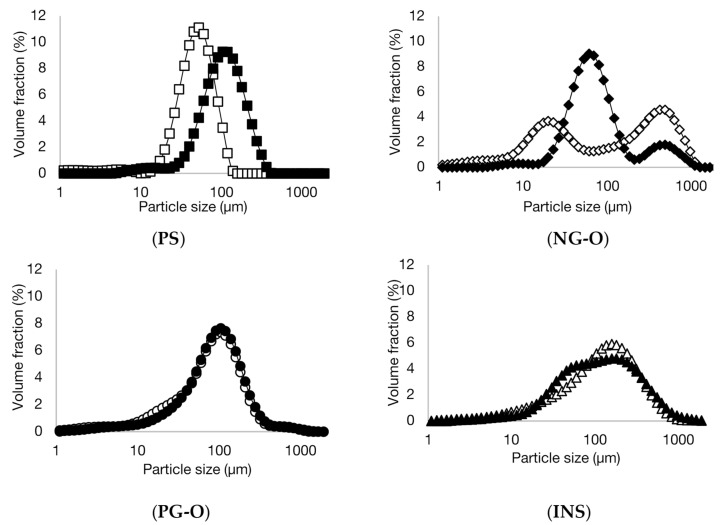
Volumetric particle size distributions of potato starch (PS), non-gelatinised open cell flour (NG-O), pre-gelatinised open cell flour (PG-O), and pre-gelatinised commercial chickpea flour (INS) suspended in cold (20 °C) (open symbols) and boiling (100 °C) (closed symbols) water.

**Figure 2:**
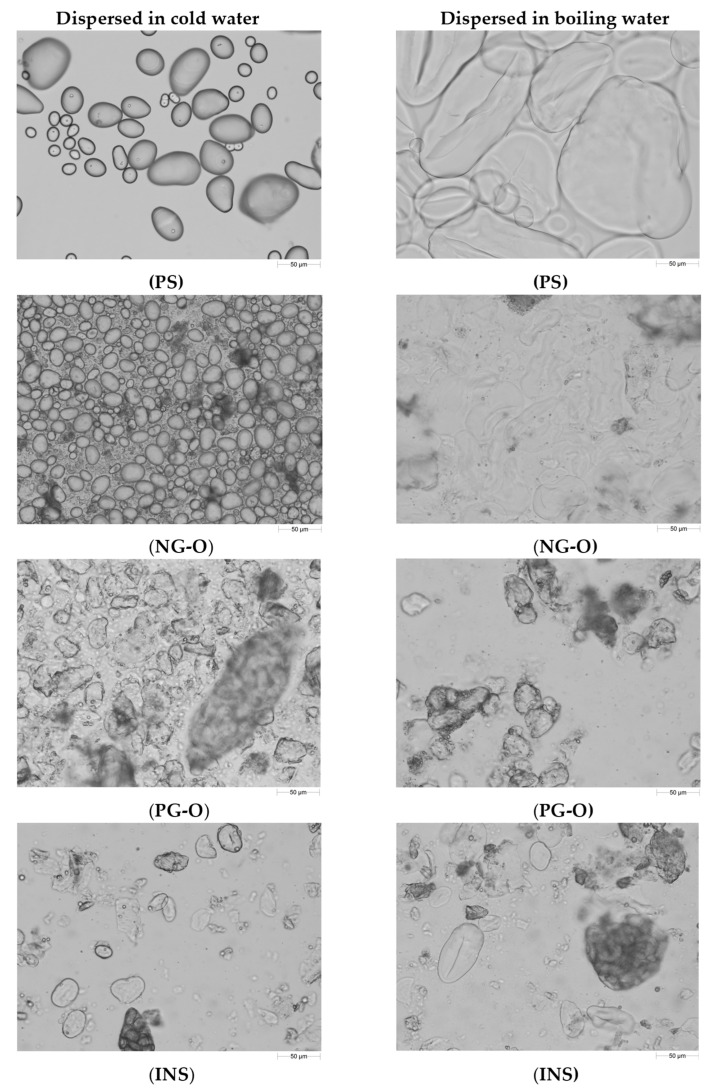
Micrographs showing the microstructure of suspensions of potato starch (PS), non-gelatinised open cell chickpea flour (NG-O), pre-gelatinised open cell chickpea flour (PG-O), and commercial pre-gelatinised chickpea flour (INS) dispersed in cold (20 °C) and boiling (100 °C) water (Magnification 20×. Scale bar: 50 µm).

In Figure 1, the particle size distribution of the different flours and PS in cold and boiling water are presented. Clearly, the non-gelatinised samples (NG-O and PS) showed different swelling behaviour compared to the pre-gelatinised samples (PG-O and INS). Micrographs of the samples are shown in Figure 2.

The PS showed a unimodal particle size distribution in both cold and boiling water, representing the native and swollen starch granules, respectively. The significant increase in particle size indicates that the PS swelled substantially in boiling water. These results were confirmed with the micrographs in Figure 2, showing a significant increase in granule size for the starch in boiling water compared to the native granules in cold water.

The NG-O showed a bimodal particle size distribution in cold water. The peak around 10–50 µm represented native starch granules, whereas the second peak around 200–1000 µm indicated the presence of aggregated cell material or fractionated cell clusters, as previously discussed by Noordraven et al. (2021) [6]. In boiling water, NG-O starch granules significantly swelled, to a particle size of 20–200 µm. Compared to PS, the NG-O had a lower swelling power.

In contrast to the non-gelatinised ingredients, the particle size distributions of the PG-O and INS samples were hardly affected by the water temperature. In both cold and boiling water, both flours showed a similar particle size distribution. This was expected, since pre-gelatinised starch is reported to possess cold-swelling properties [35]. The particle size distribution of the dispersed PG-O ranged around 10–630 µm. The tail towards smaller particles was the result of the ball milling step during the production process of the flour. From the micrographs it is observed that the fractionated swollen starch granules were present at around 20–50 µm, whereas larger particles were present in form of fractionated cell clusters or intact cells. The maximum particle size was lower for the PG-O (around 630 µm) compared to the NG-O (around 1000 µm) due to the ball milling step during production. The INS showed a similar distribution to the PG-O, however, with a broader particle size range. The maximal particle size was around 1000 µm, which indicated that the production process of the INS contained a less intensive milling step. Comparing the micrographs of PG-O and INS, similar swollen granule sizes were observed and both flours contain (partly) intact chickpea cells.

In conclusion, PS and NG-O significantly swelled in boiling water, although the NG-O granules did not expand to the same size as the swollen PS granules. Although the particle size distributions of the PS and PG-O after dispersion in boiling water were in a similar range, the PS granules swelled more strongly and the larger particles in the PG-O were mostly attributed to other cell material. INS behaved similarly to PG-O but an increased amount of larger particles were present.

### 3.2. Flow Behaviour of the Instant Soups

**Figure 3:**
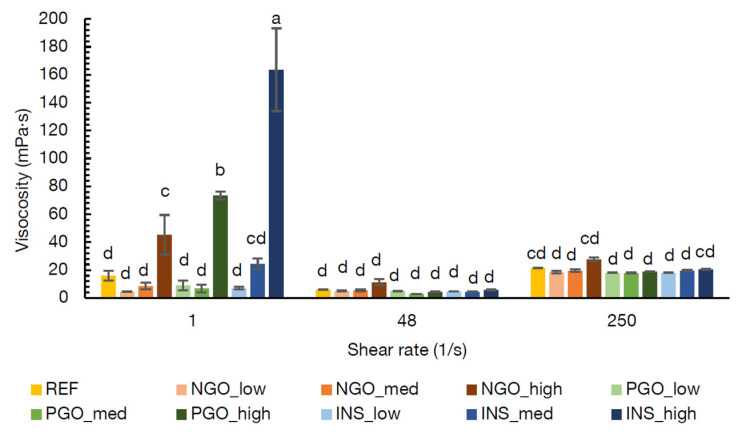
Viscosities 60 °C of instant soups containing potato starch (REF) or non-gelatinised (NGO), pre-gelatinised (PGO) or commercially available pre-gelatinised (INS) chickpea flour at low, medium (med), and high concentrations (Table 1) at different shear rates. Viscosity values indicated with the same letter are not significantly different (α = 0.05).

In Figure 3, the viscosities at 60 °C of the REF soup (at medium concentration) and the NG-O, PG-O, and INS containing soups (at low, medium, and high concentrations) are shown at different shear rates. The lowest shear rate (1 s^−1^) represented the serving condition of the soup, the shear rate of 48 s^−1^ was an approximation of the shear rate during swallowing, and the rate of 250 s^−1^ represented mixing [36,37].

Figure 3 shows that the flour concentration largely affected the viscosity at the lowest shear rate (1 s^−1^), whereas the viscosity seemed to be concentration independent at the higher shear rates (in this concentration range). This indicates that different network structures were formed in the different soups but that the critical concentration to significantly influence the viscosity was only reached at the high concentration, as no significant differences were found between the low and middle concentrations [38]. However, this network was readily broken down at a shear rate of 48 s^−1^, which represents swallowing, probably due to the alignment of particles with the flow direction [39].

Considering the viscosity during serving, swallowing, and stirring conditions, all soups containing chickpea flour were comparable to the REF soup at both medium and low flour concentrations. Since the aim of this research was to obtain an acceptable soup, while additionally improving the nutritional quality, the medium concentration of chickpea flour was chosen for further analyses. In this way, a higher nutritional improvement was obtained compared to the low flour concentration. Considering the protein contents of the chickpea flours was around 18–22% (*w*/*w*) (as analysed by Noordraven et al. (2021) [6] for NG-O and PG-O and as informed by the supplier for INS), the 1:1 replacement of PS with chickpea flour results in a 3–4% (*w*/*w*) increase in high quality protein in the soup powder. Additionally, the replacement contributes to an increased vitamin and mineral content.

**Figure 4:**
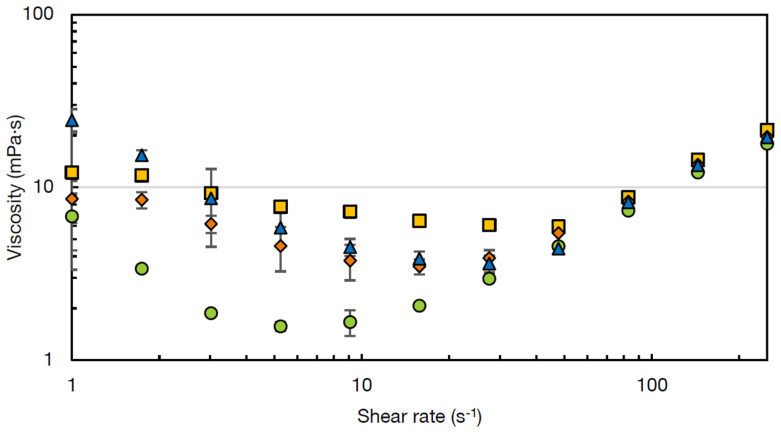
Viscosity curves at 60 °C of instant soups containing potato starch (REF) (■) or non-gelatinised (NG-O) (◆), pre-gelatinised (PG-O) (●) or commercial pre-gelatinised (INS) (▲) chickpea flour at medium concentration, showing viscosity (η) as function of shear rate ($\dot{\gamma }$).

In Figure 4, the viscosity curves at 60 °C of the soups with a medium concentration of PS or chickpea flour are given over the shear rate range 1–250 s^−1^. Two phenomena are visible, namely shear-thinning behaviour at lower shear rates and shear-thickening behaviour at higher shear rates. This type of viscosity curve was previously described for starch-water suspensions [39,40].

**Figure 5:**
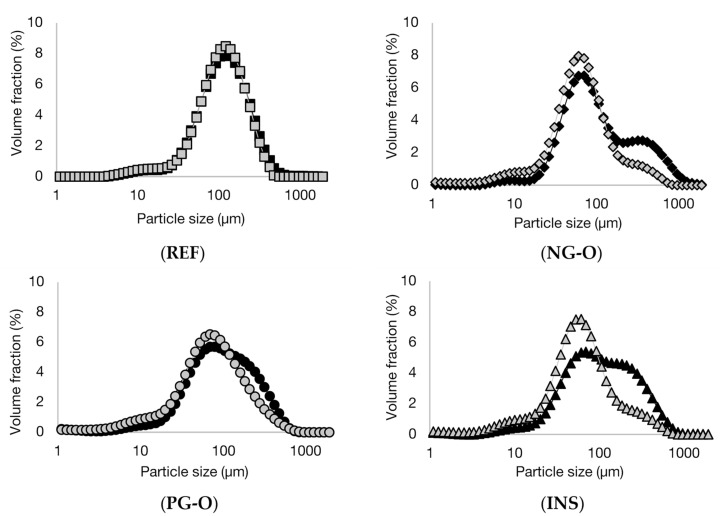
Volumetric particle size distributions of reference soup containing potato starch (REF), and chickpea flour soups containing non-gelatinised open cell flour (NG-O), pre-gelatinised open cell flour (PG-O), and pre-gelatinised commercial chickpea flour (INS) suspended in boiling water without (black symbols) and with sonication (grey symbols).

The shear thinning behaviour of the soups seems to be the lowest for the REF soup and the highest for the INS soup. Shear thinning behaviour results from particle layering with the direction of flow, making them more easily slide past each other compared to when they are randomly distributed [39]. This effect is reported to be larger when suspended particles are larger, as Brownian motion is less effective for these larger particles compared to smaller particles at the same particle density [41]. This is in line with the results for particle size distribution (Section 3.1), where INS contained the highest particle size. Secondly, shear thinning can be the result of the breakdown of particle clusters with increasing shear rate [42]. For this reason, it was hypothesised that the soups containing chickpea flours, in particular INS and PG-O flour, possessed more particle clusters compared to the reference soup. To confirm this hypothesis, the particle size distributions of the four prepared soups were analysed with and without a sonication step inside the particle size analyser. The sonication step was expected to break up shear-sensitive clusters such as protein-starch aggregates present in the soup [43]. The particle size distributions of the four soups, measured with and without sonication, are shown in Figure 5. As expected, the particle size distribution of the REF soup with PS was not influenced by the sonication, indicating that no shear-sensitive particle aggregates were present. In contrast, all three soups with chickpea flour showed a decrease in particle size, indicating the breakdown of particle clusters upon sonication. This effect was, as expected, the largest for the soup with the INS flour. 

When the critical thickening shear rate was reached, the shear thinning behaviour transitioned into a shear thickening behaviour [39]. This critical shear rate is dependent on particle size, particle concentration, and polydispersity and interactions between the particles [44]. The critical shear rate is reported to be inversely correlated to the particle size in the suspensions, which explains why the REF soup showed the highest critical stress, as it was the soup with the lowest maximum particle size (<500 µm) (Figure 5).

Suspensions of several starch types, including potato starch, have been reported to show shear thickening behaviour at increased shear [39,40,45,46]. This shear thickening behaviour is more pronounced for waxy starches compared to normal starch, as shear thickening is a property of amylopectin as opposed to amylose [45]. Viscosity increase under strong shear forces is attributed to the fact that particles are pushed into clusters under high shear, resulting in increased drag forces between the particles [39,47]. The increased resistance due to the interaction between these particle clusters results in an increased viscosity [46]. Additionally, with increased shear, the ordered particle layers, which resulted in shear thinning are disordered again, resulting in an increased viscosity [47,48].

The different soups presented a similar viscosity at increased shear rates (50–250 s^−1^). This behaviour was previously observed for different corn starch suspensions at concentrations between 10 and 30%. At higher particle concentrations >40% thickening vastly increased, due to additional thickening caused by the confining forces of the system borders [39]. However, these high concentrations of starch or chickpea flour were not relevant for the present study.

### 3.3. Flow Behaviour of the Dry Soup Powders

In Table 2, the flow behaviour parameters of the reference soup powder and chickpea-flour-containing soup powders are presented.

The bulk density of the PG-O containing soup powder was the same as the REF soup powder. However, the NG-O and INS powders had a lower and higher apparent bulk density, respectively. Hence, when INS or NG-O flour would be used in the soup powder formulation, the product volume in the single-portion packages would slightly change, which could be noticed by the consumer.

The flowability index (ff_c_) gives information on the flow behaviour of the dry powders, where a ff_c_ value below 1 indicates that the powder does not flow, a ff_c_ value between 1–2 indicates a very cohesive powder, between 2–4 a cohesive powder, between 4–10 easy-flowing powder, and a ff_c_ value above 10 a free flowing powder [28]. No significant differences were found between the four soup powder formulations, although the powders with chickpea flours are categorised as cohesive samples whereas the reference powder as very cohesive samples. This indicates that addition of chickpea flour might slightly improve the flowability of the powders.

The basic flow energy (BFE) is the energy needed to displace powder when forced to flow, for example during mixing [28]. The BFE of the REF powder and the PG-O powder did not significantly differ, whereas the NG-O and INS powders required less energy to be displaced. Therefore, the replacement of PS by chickpea flour could be advantageous.

The specific energy (SE) gives information about the flow behaviour in a low stress environment (e.g., during filling) [28]. An indication about the cohesiveness of the powders can be obtained from the SE, where an SE value below 5 indicates low cohesion, an SE value between 5–10 moderate cohesion, and an SE value above 10 a high cohesion. The REF powder showed high cohesion, whereas all chickpea containing powders showed moderate cohesion values. The replacement of PS with chickpea flour thus decreased inter-particulate interlocking and therefore could reduce the risk of blockages during filling [49]. For this reason, it could be beneficial to replace PS with chickpea flour.

The stability index (SI) of the soup powders represents the stability of the powder to repeated flow [30]. A powder with an SI between 0.9 and 1.1 has a stable powder rheology. When the SI is lower than 0.9, the powder is unstable, potentially due to de-agglomerations. In contrast, when the SI is higher than 1.1, the powder is unstable as result of de-aeration, moisture uptake, or agglomeration segregation [30,32]. There was no significant difference between the REF and chickpea-flour-containing powders. However, the PG-O and INS powders fell in the stable sample category, whereas the REF and NG-O powders were slightly unstable. These results indicated that it is not disadvantageous to replace PS with chickpea flour.

Lastly, the flow rate index (FRI) is an indicator for the sensitivity of powders to changes in flow rate. A powder with an FRI around 1 is considered to be independent to changes in flow rate, which is common for powders with large particle size distributions. Powders with an FRI between 1.5 and 3.0 show moderate sensitivity to flow rate [32]. All four soup powders had an FRI index around 1 and no significant differences were found between the FRI values of the powders. Again, this indicates that PS could be replaced with chickpea flour without inducing problems.

### 3.4. HS-SPME-GC-MS Volatile Fingerprinting

**Figure 6:**
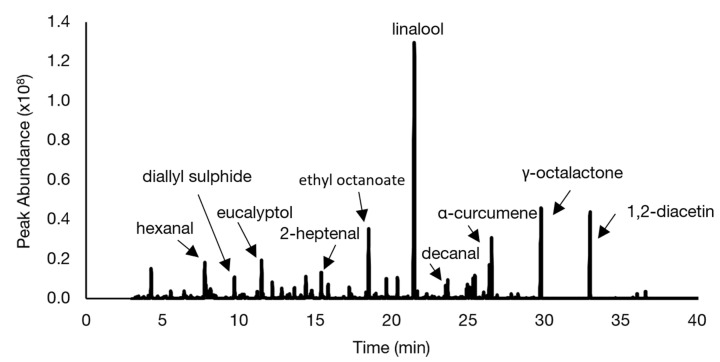
Representative total ion chromatogram of the headspace volatile fraction of the reference soup containing potato starch.

In Figure 6, a representative chromatogram for the reference soup is shown and the identities of the relevant volatiles with highest abundance are indicated. As expected, the chromatograms of the chickpea-flour-containing soups looked similar to the chromatogram of the reference soup, although differences in peak abundances were visible for specific peaks (data not shown). To obtain an overview of all differences in peak abundances between the samples, MVDA was applied.

**Figure 7:**
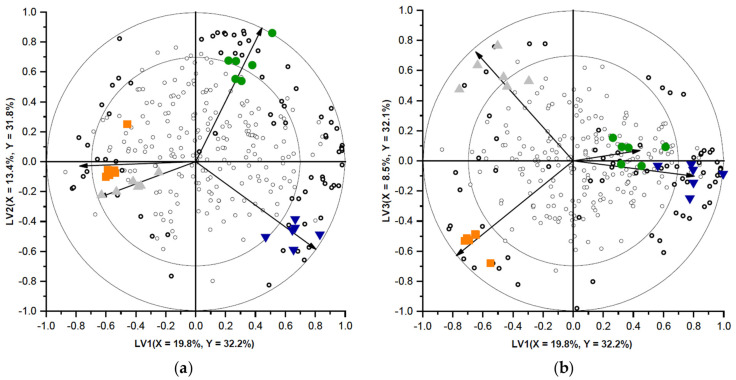
Biplot of LV1 versus LV2 (**a**) and LV1 versus LV3 (**b**) of a PLS-DA-model representing the volatile headspace components of the reference soup (●) and soups containing non gelatinised open cell chickpea flour (NG-O) (▼), pre-gelatinised open cell chickpea flour (PG-O) (▲), and pre-gelatinised commercially available chickpea flour (INS) (◼). The percentage of *X*- and *Y*-variance explained per LV is shown on the axes. Soup samples are represented as coloured symbols and volatile components are represented as open circles. Markers with |VID| > 0.7 are represented as bold circles. The vectors represent the correlation loadings for the *Y*-variables (classes). The outer and inner circles on the biplot represent the correlation coefficient of 1.0 and 0.7 respectively, indicating the area where the most important discriminating volatiles are presented.

To characterise the differences between the volatile profiles of the different soup samples, a PLS-DA model with three latent variables, explaining 96.1% of the Y-variance, was selected. In Figure 7, two biplots (LV1 versus LV2 and LV1 versus LV3) representing this PLS-DA model are shown. The different soup samples are visualised by the differently coloured objects, and all headspace volatiles present are visualised with open circles. The four different classes (representing the four different soup recipes) are clearly separated, indicating that the volatile profiles of the samples were distinct.

The correlation loadings of the classes (Y-variables) in the biplots are indicated by the vectors. The longer the vector, the better the class towards which the vector is pointed is explained by the PLS-DA model. Volatiles close to the centre of the biplot have a similar concentration in all samples, whereas volatiles represented closer to a certain class are present in higher abundance in this specific class (and in lower abundance in the opposite positioned classes) [33]. In Figure 7, many volatiles were present in the centre of the biplot. This was expected as all soups contained the same soup base, containing many flavouring compounds. The soup powders were only 16.5% (*w*/*w*) different from each other and therefore only part of the volatile composition was expected to be altered.

VID coefficients were calculated to investigate the differences between the soup samples in more detail. For the reference soup and the soups containing NG-O, PG-O, and INS chickpea flour, respectively, 18, 21, 5, and 10 discriminant compounds with a positive VID (>0.7) and 1, 6, 12, and 11 discriminant compounds with a negative VID (<0.7) were found, which are presented in Table 3. The discriminant compounds with a positive VID were present in higher abundance in the specific class, whereas the discriminant compounds with a negative VID were present in lower abundance in the specific class [34]. The volatile compounds which were present in higher abundance in the headspace of the reference soup sample were mainly terpenoids (11 out of 16 identified compounds). Additionally, two esters, two alcohols, and a hydrocarbon were present at higher concentration. Analysis of the headspace of PS dispersed in boiling water confirmed that only (*E*)-2-nonenal and dodecane were present in PS. These two compounds were present in higher concentration in the PS compared to the chickpea flours. Generally, alkanes do not significantly contribute to the aroma of foods [50,51], but 2-nonenal is known to have an unpleasant, grassy, and greasy odour [52].

All terpenoids and esters as well as the (*E*)-but-2-enal originated from the other soup ingredients. Therefore, it is suggested that these flavour compounds interacted with molecules present in the different chickpea-flour-containing soups, lowering their presence in the headspace of the respective soups. The chickpea flours contained fat, protein, and a different type of starch compared to PS. Proteins are able to bind aroma molecules, using reversible hydrophobic interactions or irreversible covalent binding [25,26]. Starch is able to bind aroma molecules by adsorption involving hydrogen bonds. Different starch types are reported to show different aroma retentions [27]. In foods, flavours are distributed between the lipid and the water phase. When the lipid content of a food is changed, the headspace volatile profile can significantly change, which could lead to an different overall perception of the flavour [26]. Terpenoids, such as α-curcumene, alloaromadendrene, γ-muurolene, and trans-calamenene, are important flavour compounds which could give rise to herbal aromas [53,54,55,56]. Possibly, the reference soup could therefore be perceived as more herbal compared to the chickpea-flour-containing soups. However, the aroma and flavour of a product are dependent on the odour thresholds, concentration, and interactions of the volatile compounds present [22,23,24].

The NG-O containing soup possessed higher concentrations of hydrocarbons, alcohols, aldehydes, terpenoids, and sulphur compounds. Of the identified compounds, 8 of the 15 were hydrocarbons, which were not considered to be very important for the aroma of the soup [50,51]. Of the other discriminant compounds, only benzothiazole, heptanal, and 1-octanol were present in the NG-O flour. Benzothiazole was the only volatile that was also present in higher concentration in the NG-O flour compared to the other chickpea flours and PS. The heptanal and 1-octanol could have resulted from (enzymatic) lipid breakdown during the flour production or soup production. Benzothiazole has been previously reported in pea flour [23]. The sulphur compounds diallyl sulphide and allyl-1-(*E*)-propenyl-disulphide originated from the other soup ingredients but appeared to be more stable in the NG-O containing soup, which could significantly influence the sensory profile of this soup [26].

The soups with the pre-gelatinised chickpea flours had a lower number of characteristic volatile compounds (discriminant compounds with positive VID). The PG-O soup contained higher concentrations of ethanol, methyl-1-butanol, 3-methylbutanal, and 2-methyl butanal. All these compounds were present in the PG-O flour and both alcohols were present in higher abundance in the PG-O flour compared to the other chickpea flours and PS. These alcohols and aldehydes probably resulted from the oxidation of lipids during the production process [57]. The soup containing INS held higher concentration of several ketones, two aldehydes, a lactone, a terpenoid, an alcohol and a hydrocarbon. All ketones, aldehydes and the alcohol were also present in higher concentration in the INS flour compared to the other chickpea flours and PS. As the supplier informed us that the INS flour production included a soaking step of the ground chickpea, these compounds could have resulted from enzymatic lipid breakdown during the flour production [58]. The γ-nonalactone and β-pinene were derived from the other soup ingredients but were adsorbed to a larger extent by other starch sources (PS, NG-O, and PG-O) compared to INS. (*E,E*)-3,5-octadien-2-one, (*E,Z*)-3,5-octadien-2-one, 3-octen-2-one, benzaldehyde, and β-pinene are reported as having earthy, woody, mushroom, resin, and turpentine-like aromas and therefore the addition of INS flour to instant soups might induce off-flavours [23,59,60,61]. However, as noted before, odour threshold, concentration, and interactions between volatiles should be considered [22,23,24].

## 4. Conclusions

In this study, the potential of chickpea flours with different microstructures as alternative thickening ingredients in instant soups was investigated. It was shown that soups with three different chickpea flours (NG-O, PG-O, and INS) reached similar viscosities values during simulated serving, swallowing, and stirring conditions, compared to a reference soup containing PS, when a 1:1 replacement of PS with chickpea flour was used. This replacement contributed to a 3–4% (*w*/*w*) protein increase in the dry powder as well as a minor increase in vitamins and minerals.

The replacement of PS with chickpea flour resulted in a slight difference in bulk density. The soup powders containing chickpea flours showed similar powder flow behaviour to the reference powder while showing easier mixing and potential reduction of blockages during filling.

Although the swelling behaviour of the PS was significantly different from that of the chickpea flours, similar flow behaviour was obtained for the soups containing different flours or PS. However, the chickpea-flour-containing soups showed higher shear thinning behaviour due to the presence of larger particles and the shear induced breakdown of particle clusters, which could possibly lead to differences in the sensory perception of the texture and mouthfeel of the soups.

It was concluded that when chickpea flours were added to instant soups, they interacted with volatile compounds such as terpenoids, potentially creating a less herbal aroma. The chickpea-flour-containing soups, depending on the flour type, contained higher concentrations of several compounds like ketones, aldehydes, alcohols, and sulphur compounds, which possibly could affect the aroma and flavour of the soups.

In conclusion, chickpea flours showed excellent potential as alternative thickening ingredient in instant soups, potentially slightly improving the powder flowability properties and the nutritional value of the soup, although some changes in volatile profile were induced. Sensory analysis is recommended to investigate the impact of the chickpea flour addition to the instant soups on the sensory attributes such as appearance, aroma, flavour, texture, and mouthfeel. Additionally, research into the use of higher concentrations of chickpea flours is recommended, to be able to improve the nutritional value to a higher extent.

## Figures and Tables

**Table 1:** Instant soup recipes for low, middle, and high concentrations of potato starch or chickpea flour.

	Reference Soup	Chickpea-Flour-Containing Soups		
Ingredients		Low c (%)	Medium c (%)	High c (%)
Potato Starch	16.51	0.00	0.00	0.00
Chickpea Flour	0.00	8.78	16.51	24.24
Maltodextrin	7.73	15.46	7.73	0.00
Sunflower Oil Creamer	23.01	23.01	23.01	23.01
Roux (70% wheat flour)	17.49	17.49	17.49	17.49
Soup Base	35.26	35.26	35.26	35.26

**Table 2:** Flow behaviour parameters for reference soup powder and soup powders containing non-gelatinised open cell chickpea flour (NG-O), pre-gelatinised open cell chickpea flour (PG-O), and commercial pre gelatinised open cell chickpea flour (INS). Values within one row indicated by the same letter are not significantly different (α = 0.05).

	REF	NG-O	PG-O	INS
Bulk Density (g/mL)	0.459 ± 0.001 b	0.423 ± 0.014 c	0.479 ± 0.005 b	0.505 ± 0.004 a
Flowability index (-)	1.72 ± 0.09 a	2.95 ± 0.58 a	2.08 ± 0.31 a	2.40 ± 0.08 a
Basic flow energy (mJ)	990.18 ± 4.68 a	763.32 ± 1.00 b	885.28 ± 65.14 ab	828.00 ± 0.02 b
Specific energy (mJ/g)	10.79 ± 0.29 a	6.07 ± 0.11 c	6.04 ± 0.37 c	9.06 ± 0.11 b
Stability Index (-)	1.17 ± 0.23 a	1.17 ± 0.21 a	0.95 ± 0.04 a	0.95 ± 0.02 a
Flow Rate Index (-)	1.18 ± 0.02 a	1.10 ± 0.00 a	1.05 ± 0.02 a	1.01 ± 0.07 a

**Table 3:** Overview of volatile headspace components in reference soup (REF) and soups containing non-gelatinised open cell chickpea flour (NG-O), pre-gelatinised open cell chickpea flour (PG-O), and pre-gelatinised commercially available chickpea flour (INS), selected with the VID procedure (│VID│ ≥ 0.7). Components with a positive VID coefficient increase during storage; components with a negative VID coefficient decrease during storage. Volatiles are ranked based on chemical class and VID coefficients. Retention index (RI) on TR-FFAP column.

**REF Soup**			**NG-O Soup**		
**VID**	**Compound**	**RI**	**VID**	**Compound**	**RI**
	*Terpenoids*			*Sulphur compounds*	
0.919	valencene ^1^	1757	0.982	benzothiazole	1963
0.886	α-curcumene	1775	0.896	diallyl disulphide	1482
0.867	β-bisabolene	1725	0.885	allyl-1-(*E*)-propenyl-disulphide	1487
0.851	α-farnesene	1751		*Terpenoids*	
0.843	alloaromadendrene ^1^	1637	0.961	calamenene	1831
0.842	(*E*)-α-bergamotene ^1^	1582	−0.776	verbenol	1609
0.790	γ-muurolene ^1^	1683		*Aldehydes*	
0.773	(*E*)-calamenene	1831	0.936	heptanal	1190
0.763	caryophyllene	1589	−0.712	2-methyl-butanal	939
0.738	γ-curcumene	1688		*Alcohols*	
0.708	(*E*)-β-famescene	1667	0.865	2-isopropyl-5-methyl-1-heptanol ^1^	1332
	*Esters*		0.724	1-octanol	1563
0.905	ethyl-dodecanoate	1847		*Hydrocarbons*	
0.848	ethyl-decanoate	1641	0.869	4,6,8-trimethyl-1-nonene ^1^	1542
	*Alcohols*		0.830	3-methyl-undecane	1170
0.722	(*E*)-2-nonenal	1538	0.811	4,6-dimethyl-dodecane ^1^	1227
0.708	(*E*)-2-butenal	1048	0.750	3,3-dimethyl-octane ^1^	998
	*Hydrocarbons*		0.748	2,6,6-trimethyl-octane ^1^	1105
0.723	undecane	1098	0.744	dodecane	1203
	*Alcohols*		0.739	7-methyl-(*E*)-4-decene ^1^	1053
−0.759	1-octen-3-ol	1455	0.731	4-methyl-decane	1002
	*Unidentified*			*Ketones*	
0.841	unidentified	-	−0.712	2-methyl-1-penten-3-one	1071
0.840	unidentified	-	−0.734	1-octen-3-one	1303
				*Unidentified*	
			0.873	unidentified	
			0.828	unidentified	
			0.790	unidentified	
			0.753	unidentified	
			0.744	unidentified	
			0.738	unidentified	
			−0.728	unidentified	
			−0.905	unidentified	
**PG-O Soup**			**INS Soup**		
**VID**	**Compound**	**RI**	**VID**	**Compound**	**RI**
	*Alcohols*			*Ketones*	
0.869	3-methyl-1-butanol	1216	0.976	(*E,Z*)-3,5-octadien-2-one	1525
0.799	ethanol	953	0.975	3-octen-2-one	1409
−0.740	1-heptanol	1460	0.930	2-heptanone	1187
−0.752	1-octanol	1563	0.841	(*E,E*)-3,5-octadien-2-one	1575
	*Aldehydes*			*Benzene derivatives*	
0.803	3-methylbutanal	942	0.911	benzaldehyde	1529
0.725	2-methylbutanal	939		*Esters*	
−0.795	(*E*)-2-butenal	1048	0.834	γ-nonalactone	2039
	*Hydrocarbons*		−0.779	ethyl-octanoate	1438
−0.707	dodecane	1203		*Alcohols*	
−0.767	2,4,6-trimethyldecane ^1^	1084	0.813	1-hexanol	1358
−0.852	2,2,4,6,6-pentamethyl-heptane	970	−0.732	2-isopropyl-5-methyl-1-heptanol ^1^	1331
−0.853	4,7-dimethyl-undecane^1^	1091	−0.746	2-nonanol	1524
−0.892	3,5-dimethyl-octane ^1^	1017		*Aldehydes*	
	*Terpenoids*		0.742	hexanal	1089
−0.711	3-carene ^1^	1140		*Hydrocarbons*	
−0.853	α-phellandrene	1158	0.726	Tridecane	1301
	*Unidentified*		−0.732	3,3-dimethyl-octane ^1^	998
0.940	unidentified	-	−0.732	4-methyl-decane	1002
−0.711	unidentified	-	−0.758	3-methyl-undecane	1170
−0.760	unidentified	-		*Terpenoids*	
			0.708	β-pinene	1096
				*Sulphur compounds*	
			−0.778	Allyl methyl disulphide	1281
				*Unidentified*	
			−0.711	unidentified	
			−0.723	unidentified	
			−0.726	unidentified	
			−0.842	unidentified	

^1^ tentatively identified.

## Data Availability

The data used for the figures are available on 10.5281/zenodo.5521400.
